# Fluoroquinolone Resistance Patterns in Multidrug-Resistant *Escherichia coli* from the Gut Microbiota of Young Children

**DOI:** 10.3390/antibiotics15020140

**Published:** 2026-01-31

**Authors:** Ludmila Suzhaeva, Svetlana Egorova, Dmitrii Polev, Alina Saitova, Daria Starkova

**Affiliations:** 1 Laboratory of Identification of the Pathogens, St. Petersburg Pasteur Institute, 197101 St. Petersburg, Russia; suzhaeva@pasteurorg.ru (L.S.); egorova@pasteurorg.ru (S.E.); 2 Laboratory of Metagenomics Research, St. Petersburg Pasteur Institute, 197101 St. Petersburg, Russia; polev@pasteurorg.ru (D.P.); saitova@pasteurorg.ru (A.S.)

**Keywords:** *E. coli*, children, multidrug-resistant *E. coli*, fluoroquinolone resistance, moxifloxacin, levofloxacin, ciprofloxacin, nalidixic acid, resistance determinants, whole-genome sequencing

## Abstract

**Background/Objectives**: The high prevalence of fluoroquinolone-resistant *E. coli* in healthy children represents a significant public-health risk, facilitating the spread of antimicrobial resistance and increasing the potential for difficult-to-treat extraintestinal infections with severe clinical outcomes. This study aimed to investigate the prevalence of fluoroquinolone resistance in multidrug-resistant *E. coli* isolated from presumptively healthy children in St. Petersburg, Russia, with a particular focus on fluoroquinolone resistance determinants. **Methods**: Phenotypic AST was performed on 307 *E. coli* isolates from fecal pediatric samples, comprising 230 isolates from 2012 to 2013 and 77 isolates from 2021 to 2022. A subset (*n* = 47) of MDR isolates underwent whole-genome sequencing. **Results**: The frequency of MDR *E. coli* strains rose significantly from 15.7% to 32.5% over the study period. The most significant increases in resistance among *E. coli* strains were to third-generation cephalosporins (CTX, CTZ) and fluoroquinolones (CIP), rising fourfold over a decade. Based on phenotypic resistance profiles of MDR *E. coli* to quinolones, the highest resistance rates were observed for MFX (80.9%) followed by NAL (74.5%), LVX (44.7%) and CIP (40.4%). Genotypic analysis revealed distinct pathways: low-level NAL resistance required only an S83 mutation in *gyrA*, whereas low-level MFX resistance was predominantly conferred by a plasmid-borne *qnr* gene. In contrast, resistance to CIP and LVX involved at least three QRDR mutations: S83L and D87N/Y in *gyrA*, and S80I in *parC*. Notably, our study showed the predominance of the ST131 and ST38 clones in *E. coli* isolated from pediatric samples. **Conclusions**: Our findings suggest that the efficacy of moxifloxacin for empirical treatment of infections caused by MDR *E. coli* might be severely compromised. Overall, the current study highlights that the pediatric gut microbiota serves as a reservoir for resistant *E. coli* with the expansion of multidrug-resistant clones independently of direct antibiotic selection pressure.

## 1. Introduction

Quinolones are a class of synthetic antimicrobial agents with a broad spectrum of antibacterial activity. The progenitor of this class, nalidixic acid, was introduced into clinical practice in the 1960s for the treatment of uncomplicated urinary tract infections caused by enteric bacteria. A pivotal advancement came with the introduction of a fluorine atom at the C-6 position of the quinolone structure, leading to the development of fluoroquinolones (FQs), which exhibit improved biological activity as well as pharmacokinetic and pharmacodynamic properties [1,2]. Currently, four generations of these drugs are in clinical use, employed against a wide range of infections, including urogenital and intra-abdominal infections, infections of the eyes, skin, soft tissues, and respiratory tract, as well as tuberculosis [3,4].

Quinolones exert their antibacterial effect by targeting two essential bacterial type II topoisomerases: DNA gyrase and topoisomerase IV, which are critical for DNA replication, transcription, and chromosome segregation. FQs trap these enzymes by forming a lethal ternary cleavage complex, comprising an antibiotic-topoisomerase-cleaved DNA complex. This complex prevents DNA religation, leading to the accumulation of these complexes in the cytoplasm and initiating pathways that result in bacterial cell death [5,6].

Bacteria have evolved several distinct mechanisms to facilitate survival in the presence of FQs. These include: (1) chromosomal mutations in the quinolone resistance-determining region (QRDR) of DNA gyrase (*gyrA*, *gyrB*) and topoisomerase IV (*parC*, *parE*) genes, which weaken drug-enzyme binding; (2) plasmid-mediated quinolone resistance (PMQR), conferred by the expression of two families of genes—*qnr* (encoding proteins that protect target enzymes) and *aac(6′)-Ib-cr* (encoding a variant of aminoglycoside acetyltransferase that acetylates norfloxacin and ciprofloxacin); (3) overexpression of efflux pumps that reduce the intracellular concentration of FQs; (4) downregulation of outer membrane porins, reducing drug influx. The accumulation of these resistance mechanisms within bacterial strain drives the evolution of high-level quinolone resistance [7,8,9].

During the first two decades of widespread FQ use, the emergence of resistance during treatment was rare. Nowadays, numerous studies have reported a steady increase in resistance to FQs among both Gram-positive and Gram-negative microorganisms worldwide [10]. Although FQs are not prescribed for patients under 18 years of age due to their potential musculoskeletal toxicity and other adverse effects, high resistance rates to these antibiotics have been reported in *E. coli* isolated from children aged 1–17, posing a potential threat for managing urinary tract infections and subsequent complications [11,12,13,14,15,16,17]. An important aspect is that the rate of fecal carriage of FQ-resistant *E. coli* in healthy individuals is often higher than that in patients with nosocomial or community-acquired infections, indicating its widespread persistence as a part of the commensal gut microbiota. For example, a study in Spain (1997) [18] found the prevalence of FQ-resistant *E. coli* to be 24% in adults and 26% in children, while resistant strains accounted for only 17% of isolates obtained from patients in clinical settings. Since the emergence of FQ resistance in children is not associated with direct fluoroquinolone therapy, the transmission of resistant isolates likely occurs through contact between adults and children within households and childcare facilities, as well as via contact with household pets and the consumption of contaminated food or water. This community-based transmission steadily expands the reservoir of resistant bacteria independent of the selection pressure on isolates from patients during treatment regimen. Thus, the high prevalence of FQ-resistant *E. coli* in healthy children represents a significant risk for the spread of antimicrobial resistance and the development of difficult-to-treat extraintestinal infections with more severe clinical outcomes.

Despite the global rise in fluoroquinolone-resistant *E. coli*, data on the prevalence and molecular mechanisms of this resistance in the gut microbiota of young children, particularly in Russia, remain scarce. This study aimed to investigate the prevalence of fluoroquinolone resistance in *E. coli* isolated from presumably healthy children in St. Petersburg, Russia, with a particular focus on fluoroquinolone resistance determinants in multidrug-resistant *E. coli*.

The current research addresses the scarcity of data regarding multidrug-resistant *E. coli* carriage among presumptively healthy young children. Here, we present a large-scale investigation of phenotypic resistance to 12 antimicrobial agents for 307 *E. coli* isolates collected from children’s gut microbiota across the periods 2012–2013 and 2021–2022. Using whole-genome sequencing, we provide a comprehensive analysis of 47 MDR isolates with particular focus on genotypic determinants of resistance to four generations of quinolones and fluoroquinolones—nalidixic acid, ciprofloxacin, levofloxacin, and moxifloxacin.

## 2. Results

### 2.1. Phenotypic Antimicrobial Susceptibility Testing (AST) of E. coli Strains

**Figure 1:**
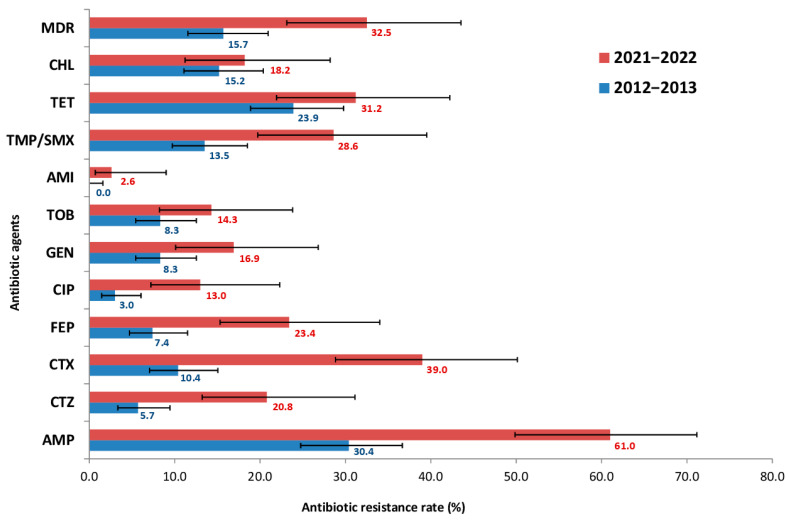
Prevalence of antibiotic resistance among 307 *E. coli* isolates from presumably healthy children, comprising 230 isolates from 2012 to 2013 and 77 isolates from 2021 to 2022. AMP—ampicillin, CTZ—ceftazidime, CTX—cefotaxime, FEP—cefepime, CIP—ciprofloxacin, GEN—gentamicin, TOB—tobramycin, AMI—amikacin, TET—tetracycline, CHL—chloramphenicol, TMP/SMX—trimethoprim/sulfamethoxazole, MDR—multidrug-resistant isolates.

Phenotypic AST was performed on 307 *E. coli* isolates from pediatric samples, comprising 230 isolates from 2012 to 2013 and 77 isolates from 2021 to 2022. The proportion of isolates resistant to each antimicrobial agent was determined using clinical breakpoints. The change in resistance frequencies over the 10-year period is shown in Figure 1.

The obtained data indicated a significant increase in *E. coli* resistance: for 9 of the 12 antibiotics tested, the frequency of resistant strains increased at least two-fold. However, the most striking increases in resistance among *E. coli* strains were to third- and fourth- generation cephalosporins (CTX, CTZ, FEP) and fluoroquinolones (CIP), rising from 10.4, 5.7, 7.4 and 3.0% in 2012–2013 to 39.0, 20.8, 23.4 and 13.0% in 2021–2022, respectively. Out of the 12 antimicrobials, the highest resistance rate was observed for AMP, which doubled over 10 years (from 30.4 to 61.0%). In contrast, the lowest resistance was to aminoglycosides (AMI). All isolates (100%) were fully susceptible to carbapenems- specifically MEM. Furthermore, the prevalence of MDR strains was also assessed. The frequency of MDR strains increased significantly over the study period, from 15.7% in 2012–2013 to 32.5% in 2021–2022.

Next, since all CIP-resistant strains were multidrug-resistant, we selected a subset of 47 MDR *E. coli* strains, comprising 19 CIP-resistant isolates, and subjected them to additional phenotypic AST against levofloxacin (LVX), moxifloxacin (MFX) and nalidixic acid (NAL). All selected strains were isolated from young children aged 1 month to 7 years old (mean age, 1 year 9 month). The MIC frequency distributions of the 47 MDR *E. coli* isolates for 15 antimicrobial agents are presented in Table S1.

Based on phenotypic AST profiles to quinolones/fluoroquinolones, the 47 *E. coli* strains were categorized into eight distinct resistance groups, designated as Groups 0–7 (Table 1). Six out of 47 isolates were susceptible to all four antimicrobial agents, whereas 19 isolates showed full resistance to all of them. Among resistant isolates, the highest resistance rates were observed for MFX (38/47, 80.9%) and NAL (35/47, 74.5%), followed by LVX (21/47, 44.7%) and CIP (19/47, 40.4%). It is worth noting that 11 of the 19 isolates (57.9%) in Group 1 were isolated from infants under one year of age (2 to 11 months).

Among the 19 FQ-resistant isolates from Group 1, all isolates (100%) were resistant to AMP and TMP/SMX, while 15 of 19 (78.9%) were resistant to TET. Notably, eight of the 19 FQ-resistant isolates (42.1%) were resistant to all of the other six antibiotic classes tested (aminopenicillins, β-lactams, cephalosporins, aminoglycosides, TMP/SMX, tetracycline, phenicol), while the remaining 11 isolates (57.9%) were resistant to at least three classes.

### 2.2. Genotypic Resistance Patterns to Quinolones/Fluoroquinolones in E. coli Strains

To investigate the mechanisms of resistance to quinolones/fluoroquinolones, whole-genome sequencing of 47 MDR *E. coli* strains was performed, followed by an investigation of the phenotypic and genotypic resistance patterns.

None of the six wild-type isolates in Group 0 had chromosomal QRDR mutations or plasmid-mediated quinolone resistance (PMQR) genes in their genomes. A total of nine point mutations in the QRDR were identified in Groups 1–7: four in the *gyrA* (S83L; S83A; D87N; D87Y), three in *parC* (S80I; E84V; E84G), and two in *parE* (I529L; S458A). The most frequent mutations were S83L in *gyrA* gene (59.6%), followed by S80I in *parC* and I529L in *parE* (38.3%, each). No mutations were found in the *gyrB* subunit of DNA gyrase. Three PMQR genes- *qnrB4*, *qnrS1* and *aac(6′)-lb-cr-* were identified in 18 FQ-resistant isolates (43.9%). Notably, the *qnrB4* gene was detected in Groups 5 and 6, whereas the *aac(6′)-lb-cr* gene was found exclusively in Group 1.

Chromosomal mutations in *gyrA* and *parC* genes were predominant in Group 1, while plasmid-mediated genes *qnrB4* and *qnrS1* were largely carried by isolates from Groups 5–7. In particular, the D87N mutation in *gyrA*, as well as E84G/N in *parC* and S458A in *parE* genes were unique for isolates in Group 1. The point mutation S80I in *parC* was present in 17/19 (89.5%) resistant strains in Group 1, whereas only one isolate from Groups 2–7 carried this mutation (*p* < 0.001).

**Figure 2:**
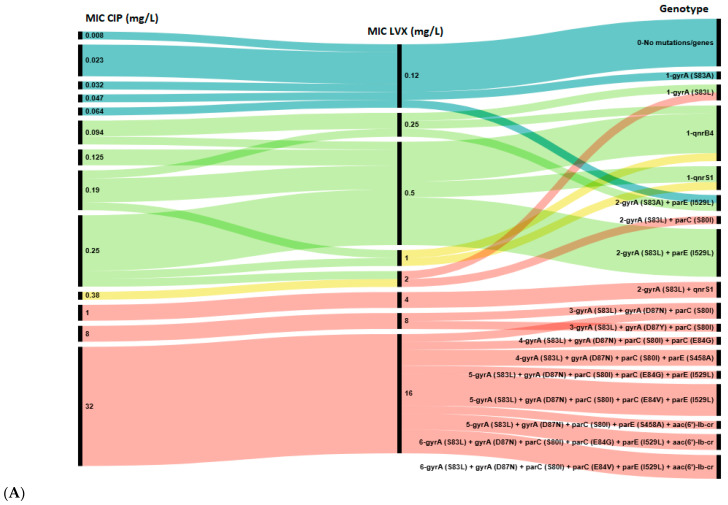

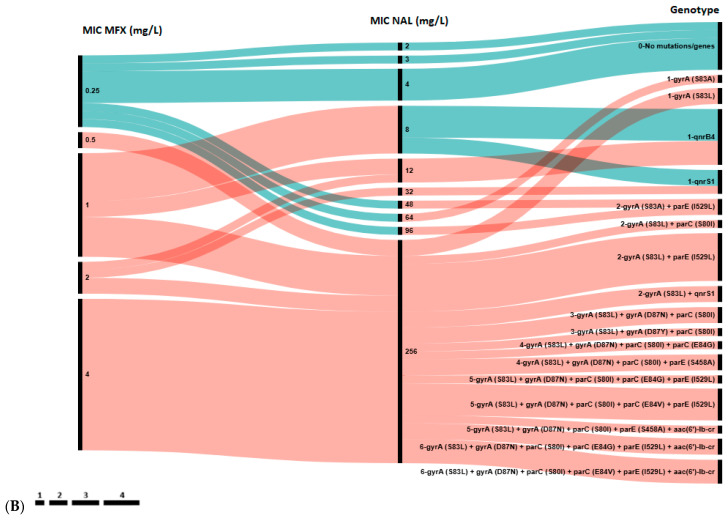
Association between genotypic and phenotypic antimicrobial susceptibility testing of *E. coli* isolates to ciprofloxacin (CIP) and levofloxacin (LVX) (**A**), and to nalidixic acid (NAL) and moxifloxacin (MFX) (**B**). The length of black lines is proportional to the number of isolates, as indicated by the scale below the figures; numbers in the MIC and Genotype rows reflect the MIC value and the number of genes in the genotype profile, respectively. Color key: Blue—wild type (WT, epidemiological cut-off value), green—susceptible strains (clinical break point), yellow—intermediate strains, red—resistant strains.

Next, we analyzed the distribution of chromosomal mutations and plasmid-borne genes against the CIP, LVX, MFX and NAL MICs for all 47 *E. coli* strains (Figure 2).

A total of 17 distinct genotypes were identified. Isolates with a single resistance determinant (either S83A in *gyrA* or *qnr* gene) showed low-level resistance to NAL (MIC 12–96 mg/L), MFX (0.5–2 mg/L), LVX (2 mg/L) and susceptibility to CIP (MIC 0.047–0.25 mg/L). However, all isolates in Group 1 with high-level resistance to all four agents were characterized by the presence of at least three mutations: S83Y, D87N/Y in the *gyrA* and S80I in *parC* gene. Notably, 10 out of 12 (83.3%) isolates in Groups 5 and 6 harbored a single *qnr* gene *(qnrB4* or *qnrS1*) without any chromosomal mutations in the QRDR. Conversely, the *aac(6′)-lb-cr* gene was found only in combination with QRDR mutations in isolates from Group 1.

Among phenotypically susceptible isolates, resistance determinants were common in isolates susceptible to CIP (21/27, 77.8%) and LVX (18/24, 75.0%), but less frequently observed in those susceptible to MFX (3/9, 33.3%). Hence, according to epidemiologic cut-off values the concordance rate between genotypic and phenotypic resistance to quinolones/fluoroquinolones achieved 95.1% for CIP and LVX, 92.7% for MFX and 85.4% for NAL. Notably, all MFX-susceptible isolates that carried resistance determinants showed resistance to NAL, and vice versa, all NAL-susceptible isolates that harbored any determinants showed resistance to MFX. Consequently, the presence of any resistance determinant showed 100% concordance with the combined MFX/NAL phenotypic resistance profiles. This observation implicitly suggests that the complementary phenotypic resistance to NAL and MFX may serve as a predictor for the presence of any genotypic resistance determinant (QRDR mutation or PMQR gene), even when phenotypic susceptibility to CIP and LVX is observed.

Overall, the obtained data demonstrate that the carriage of multiple resistance determinants in the QRDR was associated with stronger phenotypic resistance: the higher the MIC value, the more resistance determinants were detected in *E. coli* isolates. Notably, five strains in Group 1, exhibiting high-level resistance, harbored two most extensive resistance profiles, each comprising six resistance determinants. Four of these strains were isolated from infants aged 2 to 5 months.

### 2.3. E. coli Molecular Type Identification and Distribution of Genotypic Resistance Patterns in E. coli Sequence Types

According to WGS analysis of 47 *E. coli* isolates, 7 phylogenetic groups, 16 sequence types, 21 serotypes, and 12 *fimH* subclones were identified. The B2/ST131/O25:H4/*fimH30* and D/ST38/O153:H30/*fimH5* lineages were predominant (19.1 and 23.4%, respectively).

**Figure 3:**
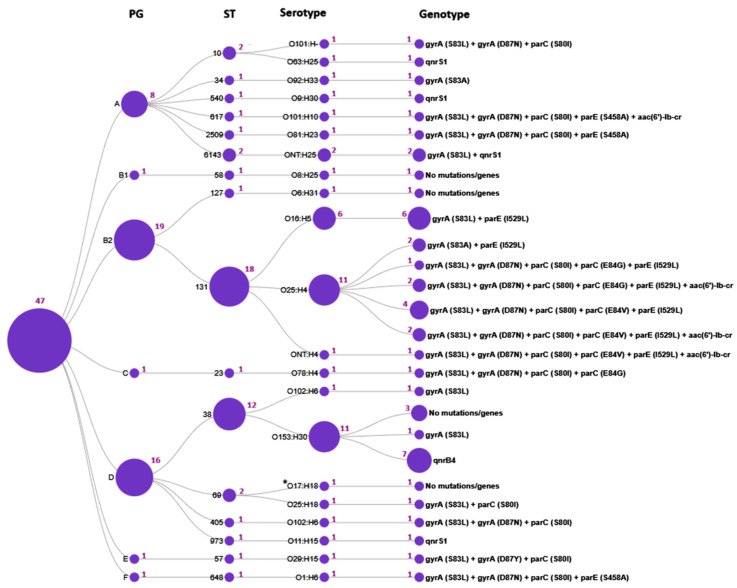
Distribution of genotypic resistance profiles across *E. coli* molecular types. The circles represent the determined sequence types (ST), phylogenetic groups (PG), serotypes, and the genotype profiles, respectively; the purple numbers adjacent to the circles indicate the number of isolates; *O17:H18 denotes an unspecified O17/O44/O73/O77/O106:H18 antigen.

We further investigated the distribution of genotypic resistance profiles across the *E. coli* molecular types (Figure 3,Table 2).

The current study has demonstrated that certain mutations and quinolone resistance genes were strongly associated with particular *E. coli* STs. Thus, ST131 exhibited the most diverse genotypic resistance profile, characterized by two to five mutations in the QRDRs and the presence of the *aac(6′)-lb-cr* PMQR gene; none of the ST131 isolates carried *qnr* genes. Intriguingly, the E84V in *parC* and I529L in *parE* mutations were identified exclusively in ST131 isolates and were absent in all other 15 STs. In contrast, ST38 isolates were typically characterized by the presence of the *qnrB4* gene. Resistance determinants in other STs were represented by one to four mutations in the QRDR or the presence of PMQR genes.

According to *fimH* subtyping, three subclones were identified within the ST131 population: *fimH30*, *fimH27* and *fimH41*. Among them, the ST131/O25:H4/*fimH30* subclone (accounting for 10 CIP- and NAL-resistant strains) was strongly associated with fluoroquinolone resistance, whereas the ST131/O16:H5/*fimH41* and ST131/O25:H4/*fimH27* subclones were fluoroquinolone-susceptible and comprised six and two CIP-susceptible but NAL-resistant isolates, respectively.

### 2.4. Association Between Efflux Pump Genes and Antimicrobial Resistance in E. coli Strains

Since multidrug antibiotic resistance is often associated with efflux pumps, we evaluated the presence of efflux pump genes in the 47 *E. coli* MDR strains and analysed the distribution of these genes in isolates of different groups (Group 0–Group 7) and sequence types. As a result, we found four types of efflux proteins AcrF, MdtM, EmrD, EmrE belonging to three families: RND (Resistance-Nodulation-cell Division family), MFS (Major Facilitator Superfamily), and SMR (Small Multidrug Resistance family). At the same time, no efflux pump genes (*qepA*, *oqxA/B*) known to confer resistance to fluoroquinolones were detected.

All 47 strains harbored both AcrF and EmrE. In contrast, EmrD and MdtM were detected in 76.6% (36/47) and 66.0% (31/47) of isolates, respectively. The presence of EmrD was significantly associated with sequence type, being found in all (100%) *E. coli* ST38 and ST131 isolates but in only 35.3% of other STs (ꭓ^2^ = 25.343, df = 2, *p* < 0.001). Similarly, MdtM was prevalent in ST131 (88.9%) and other STs (88.2%), however was absent in all ST38 isolates (ꭓ^2^ = 31.223, df = 2, *p* < 0.001).

**Figure 4:**
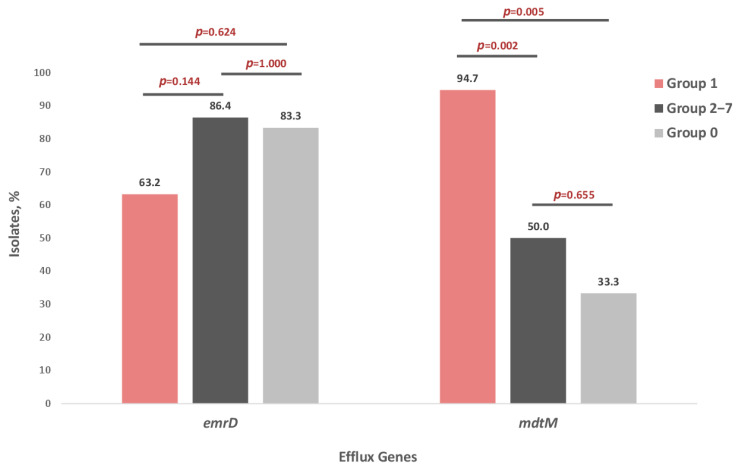
Distribution of EmrD and MdtM efflux pump genes in seven groups of *E. coli* strains: Group 1—isolates resistant to four quinolones—NAL, CIP, MFX, LVX (*n* = 19), Groups 2–7—isolates resistant to one to three quinolones (*n* = 22), Group 0—isolates susceptible to four quinolones—NAL, CIP, MFX, LVX (*n* = 6).

Analysis of EmrD and MdtM distribution among FQ-resistant *E. coli* isolates revealed that the frequency of the EmrD efflux gene did not differ significantly between groups. In contrast, the frequency of the MdtM gene was significantly higher in Group 1 compared with both Groups 2–7 and Group 0 (*p* < 0.05) (Figure 4).

## 3. Discussion

Antimicrobial resistance represents a paramount global health challenge, prioritized by the World Health Organization (WHO) as one of the top ten global public health concerns [19]. Driven primarily by antibiotic overuse, AMR has led to the emergence and spread of multidrug-resistant (MDR) bacteria, thereby contributing to the worldwide resistance crisis and undermining existing programs for infection prevention and control. Our study calls attention to the reservoir of MDR *E. coli* in presumptively healthy young children with a specific focus on the fluoroquinolone resistance patterns. Although fluoroquinolone usage is strictly limited in pediatric patients, the emergence and prevalence of resistant strains are abundant and have increased substantially over the past decade. According to previous reports, the carriage of quinolone-resistant *E. coli* in children reached 9.3% in Poland, 22.0% in Spain, 17.6% in China, 52.0% in India, 31.1% in Iran, and 26.9–67.7% in Vietnam [20,21,22,23,24,25,26].

This study provides the first comprehensive investigation of antibiotic resistance patterns in *E. coli* collected from children’s gut microbiota across 2012–2013 and 2021–2022 in St. Petersburg, Russia. It is worth noting that the 2021–2022 collection period coincided with strict COVID-19 lockdown measures, which accounts for the smaller sample size (*n* = 77) compared to the prior cohort size (*n* = 230). Nevertheless, over this decade, we observed a two-fold increase in the prevalence of MDR *E. coli*, alongside a dramatic fourfold rise in resistance to both fluoroquinolones and cephalosporines. It is worth noting that the COVID-19 pandemic in 2020–2021 triggered a sharp rise in levofloxacin use in Russia. A systematic review indicates that roughly 70% of COVID-19 patients in both outpatient and hospital settings were prescribed systemic antibiotics, predominantly fluoroquinolones (2.82 DID* in 2019 vs. 3.86 DID in 2020; *defined daily dose (DDD) per 1000 inhabitants per day (DID)). Levofloxacin consumption, in particular, nearly doubled from 0.64 DID in 2019 to 1.33 DID in 2020. Thus, consumption of fluoroquinolone antibacterial drugs increased by 210% over this period [27,28].

Overall, these alarming trends in resistance to high-priority antimicrobial agents severely complicate the management of community-acquired infections and the development of subsequent complications, posing a protentional risk of fatal outcomes for children under one year of age. Moreover, the presence of MDR *E. coli* in healthy children affects the composition of commensal microbiota, which may acquire resistance genes and act as a reservoir of bacterial resistance, potentially becoming the dominant population even in the absence of antibiotic selective pressure. While antimicrobial stewardship is crucial to reduce antibiotic overuse, a study by Tchesnokova et al. [29] suggests that merely decreasing antimicrobial prescriptions (ciprofloxacin, in particular) was insufficient to curb the community spread of resistant *E. coli*, which continued to increase and develop co-resistance. This finding indicates that supplemental measures, such as the identification of carriers, may also be necessary.

The factors driving the emergence and transmission of MDR isolates in a community setting are multifactorial, encompassing changes in antibiotic use and treatment strategies, bacterial transmission dynamics and microbial evolution. However, the detection of fluoroquinolone-resistant *E. coli* strains in fluoroquinolone-naïve children suggests that resistance is likely acquired from external sources, such as from the mother during childbirth, the hospital environment, contact with family members or pets, or via contaminated water and food [30,31,32]. Numerous studies have shown that *E. coli* from healthy children was more frequently resistant to fluoroquinolones compared to *E. coli* from children with diarrhea, and also that young children have the highest risk of carrying antibiotic-resistant commensal bacteria in community settings [24,33].

*E. coli* is a highly genetically diverse microorganism with a significant capacity for evolution through gene modification and acquisition. Crucially, *E. coli* employs all known mechanisms to develop resistance, such as limiting drug uptake, modifying drug targets, inactivating drugs, and active drug efflux [34]. In the case of quinolone resistance, the primary mechanism involves chromosomal mutations in the highly conserved target region QRDR of DNA gyrase and topoisomerase IV. Secondary mechanisms, such as plasmid-mediated quinolone resistance (PMQR) genes (with the *qnrS1* being the most effective against FQs) and efflux pump overexpression, also contribute to resistance development [35,36].

While single *gyrA* mutations often confer resistance to nalidixic acid, resistance to later-generation fluoroquinolones (CIP, LVX, MFX) typically requires multiple mutations. Surprisingly, our findings revealed the highest resistance rate for the fourth-generation agent MFX (80.9%), followed by NAL (74.5%), LVX (44.7%) and CIP (40.4%). Moreover, while the initial S83L/A chromosomal mutation in *gyrA* was sufficient to confer low-level resistance to NAL, resistance to MFX was more frequently associated with the presence of plasmid-borne *qnr* genes. Our data suggest that despite its newer-generation status, MFX has an unexpectedly low genetic barrier to resistance in *E. coli*. This suggests that the antibacterial activity of moxifloxacin against *E. coli* is vulnerable, as the presence of a single PMQR gene can compromise its efficacy.

In contrast, resistance to CIP and LVX still develops through a multistep evolutionary process, involving the accumulation of multiple QRDR mutations. According to the current results, isolates that have evolved fluoroquinolone resistance (CIP) harbored at least three mutations: S83L and D87N/Y in *gyrA*, and S80I in *parC*. Although the S83L mutation in *gyrA* is widely recognized as conferring high-level resistance to ciprofloxacin and levofloxacin, our study found no association between this mutation and resistance to CIP and LVX, while the D87 mutation emerged as a more critical determinant for fluoroquinolone resistance. Furthermore, our findings underscore the critical role of a combination of the D87 mutation in *gyrA* and the S80I in *parC* in the development of CIP and LVX resistance, suggesting a synergistic effect in driving fluoroquinolone resistance development. Intriguingly, Teichmann et al. demonstrated that a set of mutations in the QRDR confers high-level resistance to fluoroquinolones with minimal overall fitness cost, highlighting the vital role of genetic background and epistatic interactions in the evolution of fluoroquinolone resistance [37]. Thus, accumulating evidence strongly suggests that the fitness advantage conferred by these specific mutations appears to have been crucial, not only for the global dissemination of MDR lineages but also for their circulation within communities even in environments with no fluoroquinolone use, enabling them to spread within healthcare settings [38,39].

Besides target chromosomal mutations, our results demonstrate that plasmid-borne genes contribute to phenotypic profile of 2nd and 3rd generation fluoroquinolones. While single *qnrB4* and *qnrS1* genes were found exclusively in CIP- and LVX-susceptible isolates, the *aac(6′)-lb-cr* gene was detected only in isolates with high-level CIP and LVX resistance and always in combination with at least four QRDR mutations. Previous studies have shown that the *aac(6′)-lb-cr* acetyltransferase variant substantially facilitates the selection of mutations in DNA gyrase and topoisomerase IV under ciprofloxacin exposure [40,41,42]. Thus, the acquisition of *aac(6′)-lb-cr* PMQR gene along with a set of QRDR mutations leads to heightened fluoroquinolones resistance. Moreover, plasmid-borne resistance genes not only contribute to an additive effect that increases the MIC value, but also expand the diversity and abundance of the gut resistome [43,44]. Consequently, the rising prevalence of MDR *E. coli* in children’s microbiota increases the risk of horizontal gene transfer to other gut commensals and pathogens, potentially compromising future therapeutic options.

Efflux pumps represent one of the most ubiquitous and active transporter families in *E. coli*, capable of conferring multidrug resistance by extruding a broad spectrum of antimicrobial agents [35]. In this study, four efflux proteins AcrF, MdtM, EmrD, EmrE were identified. Among these, the AcrF and EmrE pumps were ubiquitous, present in all 47 *E. coli* isolates, whereas the prevalence of MdtM was significantly higher in isolates resistant to all four quinolone agents: NAL, CIP, LVX and MFX. The AcrF is a component of the AcrEF-TolC multidrug efflux pump, which is typically silent or weakly expressed. However, when activated under stress conditions, AcrF in cooperation with MdtM can form a well-coordinated transport system for the expulsion of antimicrobial compounds [45]. We also observed that the prevalence of the MdtM efflux pump was lineage-dependent: MdtM was present in the majority (88.9%) of ST131 *E. coli* but absent in all ST38 isolates. This finding suggests that the ST131 clone employs distinct evolutionary pathways and genetic strategies for developing multidrug resistance: the stress-responsive induction of MdtM together with AcrEF-TolC may confer a selective advantage that contributes to the global dominance of the multidrug-resistant ST131 clone.

Our data indicate that the dominant clone was ST131—the most successful pandemic fluoroquinolone-resistant clone worldwide and a leading cause of urinary tract infections. Notably, the dominant resistant lineage varies geographically: ST1193 is predominant in China, while ST692 is more common in Nigeria [21,46].

Furthermore, we observed significant lineage-specific divergence in the prevalence of resistance determinants. The exclusive presence of *parC* E84V and *parE* I529L mutations in ST131 isolates may suggest a particular adaptive evolutionary pathway for this clone. As the I529L mutation in the *parE* gene is relatively rare, its emergence likely represents a later step in fluoroquinolone resistance development. Gradual accumulation of mutations—first in *gyrA*, then *parC* and finally *parE*—appears compatible with the ST131 genomic background, enabling high-level resistance without significant fitness costs. This evolutionary trajectory aligns with the reconstruction of evolutionary events by Ben Zakour et al. [47], who demonstrated that fluoroquinolone resistance mutations in ST131 emerged alongside the antibiotic’s clinical introduction in North America post-1986, facilitating the clone’s rapid global expansion. However, they note that a critical prerequisite for this success was the prior acquisition of virulence and colonization factors, which occurred before the emergence of *gyrA* and *parC* mutations. The authors describe this convergence of events as a “perfect storm” for multidrug-resistant pathogen evolution.

In contrast, ST38 isolates were typically characterized by the presence of the *qnrB4* plasmid gene. This observation may indicate a greater initial dependence on horizontal gene transfer for low-level resistance with slower accumulation of chromosomal QRDR mutations, possibly due to higher fitness constraints or different ecological pressures.

We acknowledge several limitations in this study. First, all samples were isolated from children in St. Petersburg and do not represent other regions, so the obtained results may not fully reflect the overall resistance patterns in Russia. Second, among the 47 MDR isolates, we identified 16 sequence types, creating an imbalance that limits robust statistical comparisons of resistance patterns across all lineages.

## 4. Materials and Methods

### 4.1. Sample Collection, Selection of Bacterial Isolates, Bacterial Isolation, and Antimicrobial Susceptibility Testing

A collection of 307 *E. coli* strains isolated from the gut microbiota of conditionally healthy children (aged 1 month to 17 years) at the St. Petersburg Pasteur Institute, was used in this study. The collection comprised 230 isolates from 2012 to 13 (mean age: 2 years 10 months) and 77 isolates from 2021 to 22 (mean age: 3 years 7 months). Participants had not received antimicrobial therapy for at least one year prior to enrollment. Children were recruited during either a routine pediatric visit (well-child checkup) or standard clinical and laboratory investigations at the Medical Center of the St. Petersburg Pasteur Institute. All participants (or their legal guardians) provided signed informed consent to participate.

The strains were isolated on Endo-agar medium and identified to the species level using routine biochemical tests and matrix-assisted laser desorption/ionization time-of-flight (MALDI-TOF) mass spectrometry with an Autof MS1000 (Autobio Labtec Instruments Co., Ltd., Zhengzhou, China). Of the initial 307 strains, 47 multidrug-resistant *E. coli* isolates were selected. Among these, 41 were resistant to at least one quinolone (NAL, CIP, LVX, MFX) as well as to at least one antibiotic from the highest-priority therapeutic classes (third-/fourth-generation cephalosporins and aminoglycosides), while the remaining six fully susceptible strains were used as controls. All 47 strains were isolated from children aged 1 month to 7 years.

### 4.2. Phenotypic Antimicrobial Susceptibility Testing (AST)

Phenotypic antimicrobial susceptibility testing for 307 *E. coli* strains was performed by the disk diffusion method against 13 antimicrobial agents: ampicillin (AMP) 10 µg, ceftazidime (CTZ) 10 µg, cefotaxime (CTX) 5 µg, cefepime (FEP) 30 µg, meropenem (MEM) 10 µg, ciprofloxacin (CIP) 5 µg, gentamicin (GEN) 10 µg, tobramycin (TOB) 10 µg, amikacin (AMI) 30 µg, tetracycline (TET) 30 µg, chloramphenicol (CHL) 30 µg, and trimethoprim/sulfamethoxazole (TMP/SMX) 1.25/23.75 µg. Interpretation of susceptibility and resistance was performed according to the European Committee on Antimicrobial Susceptibility Testing (EUCAST, 2025) clinical breakpoint interpretation [48]. *E. coli* isolates that demonstrated resistance to three or more different classes of antimicrobials were assigned as multidrug resistant (MDR).

The susceptibility of the 47 strains to CIP and to the additional agents MFX and LVX agents was studied using the serial broth dilution method with an Automated Microorganism Identification and Antimicrobial Susceptibility Testing Analyzer AutoMic-i600 (Autobio Labtec Instruments Co., Ltd., Zhengzhou, China). In parallel, the minimum inhibitory concentrations (MICs) of nalidixic acid (NAL) and CIP for the 47 *E. coli* strains were determined by the gradient diffusion method using E-tests (bioMérieux, Marcy-l’Étoile, France) and Mueller Hinton agar (Condalab, Madrid, Spain). Strain phenotypes wild-type (WT) or non-wild-type, (NWT) were assigned by comparing the MICs to the epidemiological cut-off (ECOFF) values provided by EUCAST (NAL-8 mg/L, CIP-0.06 mg/L, LVX-0.125 mg/L, MFX-0.25 mg/L).

### 4.3. DNA Extraction, Whole-Genome Sequencing (WGS) and Bioinformatics Analysis

WGS was performed on the 47 quinolone-resistant multidrug-resistant *E. coli* isolates. Genomic DNA was extracted using the QIAamp DNA Mini Kit (QIAGEN GmbH, Hilden, Germany) according to the manufacturer’s guidelines. The DNA concentration was quantified on a Qubit 4.0 fluorometer. DNA libraries were constructed via enzymatic fragmentation using the Fragmentation Through Polymerization (FTP) method, followed by ligation of universal adapters and PCR-based barcoding using the Universal Adapter (MDI) Module (Nanodigmbio Biotechnology Co., Ltd., Nanjing, China), as described previously [49]. Libraries were sequenced on a DNBSEQ-G50 platform (MGI Tech Co., Shenzhen, China). Paired-end sequencing was performed, yielding 2 × 150 bp reads.

The raw paired-end reads were analyzed using FastQC software (v.0.12.1; Babraham Institute, Cambridge, UK), then trimmed by Trim Galore! (Version 0.6.7; Babraham Institute, Cambridge, UK) and assembled de novo by the SPAdes assembler software (version 3.13.1; St. Petersburg State University, St. Petersburg, Russia). The assembled genome sequences for the 47 isolates were deposited in the NCBI GenBank under the project number PRJNA1386987: Genetic sequencing for surveillance of Multidrug-Resistant *Escherichia coli* in Pediatric Gut Microbiota.

The obtained sequences were analyzed for antimicrobial resistance genes (ARGs) detection using the ResFinder 4.5.0 and AmrFinder Plus platforms. The molecular typing of *E. coli* isolates was performed using MLST 2.0, SerotypeFinder 2.0, Ectyper 2.0.0, and CHTyper 1.0 with default parameters.

### 4.4. Statistical Analysis

Statistical analysis was performed using the SPSS 13 software package. We used Pearson’s chi-square (χ^2^) test and Fisher’s Exact Test to determine the significance of differences. The 95% confidence intervals for proportions and frequencies were calculated using the Wilson method.

## 5. Conclusions

This study presents the first investigation of antibiotic resistance patterns with a particular focus on fluoroquinolone resistance in *E. coli* from the gut microbiota of presumptively healthy children in St. Petersburg, Russia, spanning the periods 2012–2013 and 2021–2022. Over this decade, we observed a drastic increase in the prevalence of multidrug-resistant *E. coli* alongside rising fluoroquinolones and cephalosporins resistance. These trends significantly impede the management of community-acquired infections and represent a potential threat of severe complications and fatal outcomes for infants under one year of age.

Among quinolone-resistant strains, the highest resistance rate was demonstrated for moxifloxacin, frequently associated with a single plasmid-borne *qnr* gene. This suggests a surprisingly low genetic barrier to moxifloxacin resistance, potentially compromising its empirical use against circulating MDR *E. coli*. In contrast, resistance to levofloxacin and ciprofloxacin develops through multiple mutations in the QRDR. The synergistic combination of *gyrA* (S83L, D87N/Y) and *parC* (S80I) was identified as critical for high-level resistance to both agents. Rare mutations in *parC* (E84V, E84G) and *parE* (I529L, S458A) were identified in isolates exhibiting the highest resistance levels. Crucially, the current study revealed lineage-specific divergence in the prevalence of resistance determinants: the *parC* E84V and *parE* I529L mutations were exclusive to the high-risk clone ST131, whereas the ST38 clone was typically characterized by the presence of the *qnrB4* plasmid gene. These results underscore the distinct evolutionary trajectories and ecological adaptations of these clones.

Overall, our findings highlight that the pediatric gut microbiota serves as a reservoir for resistant *E. coli* with the expansion of multidrug-resistant high-risk clones independently of direct antibiotic selection pressure. The high prevalence of fluoroquinolone-resistant *E. coli* in healthy children calls for enhanced ongoing surveillance of antimicrobial resistance in commensal populations as a key preventive strategy to guide antibiotic stewardship programs and restrain the further evolution and spread of resistance.

## Figures and Tables

**Table 1:** Phenotypic resistance profiles to quinolones/fluoroquinolones for the 47 MDR *E. coli* strains. Resistant (R; Red); Intermediate (I; Yellow); Susceptible (S; Green); MFX—moxifloxacin; LVX—levofloxacin; CIP—ciprofloxacin; NAL—nalidixic acid.

Antimicrobial Agent				Number of Isolates, (*n* = 47) N (%)	Groups
MFX	LVX	CIP	NAL		
S	S	S	S	6 (12.8)	Group 0
S	S	S	R	3 (6.4)	Group 7
R	S	S	S	6 (12.8)	Group 6
R	S	S	R	9 (19.1)	Group 5
R	I	S	R	2 (4.3)	Group 4
R	R	S	R	1 (2.1)	Group 3
R	R	I	R	1 (2.1)	Group 2
R	R	R	R	19 (40.4)	Group 1

**Table 2:** Frequency of QRDR mutations and PMQR genes among *E. coli* sequence types (STs).

QRDR Mutations/PMQR Genes	Number of *E. coli* Isolates, N (%)				*p*-Value
	Total (*n* = 47)	ST38 (*n* = 12)	ST131 (*n* = 18)	Other STs (*n* = 17)	
No mutations/genes	6 (12.8)	3 (25.0)	0 (0)	3 (17.6)	0.1
*gyrA* (S83L)	28 (59.6)	2 (16.7)	16 (89.9)	10 (58.8)	<0.001
*gyrA* (S83A)	3 (6.4)	0 (0)	2 (11.1)	1 (5.9)	0.473
*gyrA* (D87Y)	1 (2.1)	0 (0)	0 (0)	1 (5.9)	0.406
*gyrA* (D87N)	16 (34.0)	0 (0)	10 (55.6)	6 (35.3)	0.007
*parC* (S80I)	18 (38.3)	0 (0)	10 (55.6)	8 (47.1)	0.006
*parC* (E84G)	4 (8.5)	0 (0)	3 (16.7)	1 (5.9)	0.246
*parC* (E84V)	7 (14.9)	0 (0)	7 (38.9)	0 (0)	0.001
*parE* (I529L)	18 (38.3)	0 (0)	18 (100)	0 (0)	<0.001
*parE* (S458A)	3 (3.4)	0 (0)	0 (0)	3 (17.6)	0.059
*qnrB4*	7 (14.9)	7 (58.3)	0 (0)	0 (0)	<0.001
*qnrS1*	5 (10.6)	0 (0)	0 (0)	5 (29.4)	0.007
*aac(6′)-lb-cr*	6 (12.8)	0 (0)	5 (27.8)	1 (5.9)	0.047

## Data Availability

All data of this study are presented in the article. The assembled genome sequences for all isolates are available under NCBI BioProject number PRJNA1386987: Genetic sequencing for surveillance of Multidrug-Resistant *Escherichia coli* in Pediatric Gut Microbiota.

## Supplementary Materials

The following supporting information can be downloaded at https://www.mdpi.com/article/10.3390/antibiotics15020140/s1, Table S1: MIC frequency distributions for 47 MDR *E. coli* isolates from young children (2012–2013 (*n* = 26) and 2021–2022 (*n* = 21)).

## Abbreviations

The following abbreviations are used in this manuscript:

WGS	Whole-genome sequencing
FQs	Fluoroquinolones
AST	Antimicrobial susceptibility testing
WT	Wild-type
AMP	Ampicillin
CTZ	Ceftazidime
CTX	Cefotaxime
FEP	Cefepime
MEM	Meropenem
NFT	Nitrofurantoin
NAL	Nalidixic acid
CIP	Ciprofloxacin
MFX	Moxifloxacin
LVX	Levofloxacin
TET	Tetracycline
MDR	Multidrug resistance
CHL	Chloramphenicol
TMP/SMX	Trimethoprim/Sulfamethoxazole
GEN	Gentamycin
TOB	Tobramycin
AMI	Amikacin
